# Supplementary material for: Maximizing NMR Sensitivity: A Guide to Receiver Gain Adjustment
Source: NMR Biomed. 2025 May 7;38(6):e70046. doi: 10.1002/nbm.70046 (PMC12059252; doi:10.1002/nbm.70046)
Supplement: Supplementary file 1 — Figure S1 Magnitude 13C (bottom) NMR free induction decay of hyperpolarized [1‐13C]pyruvate (96 mM) at 1 T, where signals > ≈380 a.u. were not adequately recorded. Signal plotted as recorded by standard NMR spectrometer in the rotation frame of the excitation pulse; small oscillations are caused by off‐resonances. The rapid decay and rising of the signal are likely caused by dynamic adjustments by the spectrometer which limits the signal to avoid overflow: This behaviour was observed in several experiments, when overflow was achieved. The signal overflow in this figure was achieved using 96 mM hyperpolarized sample with a 66° pulse and RG of 31 dB. The maximum value achieved was 384.2 a.u. Figure S2 Constant signal experiment on the 15N‐sample at 9.4 T showcases the linearity of the signal and calibration of the 90° pulse. The signal gets noisier for higher RGs, because the flipping angle gets as low as 0.142° at RG 101. It also demonstrates that for a given threshold (here signal ≈ 0.85·105 a.u.) one should maximise first the flipping angle and then RG if maximum SNR is the goal. It is apparent, similar to Figure 3‐15N from the main text, that the signal is higher for lower RG values if sin(α)·RG is kept constant. In theory, the signal should stay constant if the RG is linear, however here, it decreases from 0.85·105 a.u. (RG 0.25) to 0.6·105 a.u. (RG 45.2), a decrease by 30%. Figure S3 Signal (left column), corresponding SNR (right column), 1H and 13C reference samples as a function of RG at 7 T using the Avance NEO console. The reference sample composition is given in the methods section. Note the maximum and a dip in (34𝑅34𝑅𝐺) after 34𝑅𝐺𝑅34𝑅𝐺𝑅𝐺=16 and the prolonged rate of SNR recovery for the 13C sample. Figure S4 Signal (left column), corresponding SNR (right column), 1H and 13C reference samples as a function of RG at 11.7 T using the Avance NEO console. The reference sample composition is given in the methods section. Note the maximum SNR is reached around R [file NBM-38-e70046-s001.pdf]

Supporting information for:

Maximizing NMR Sensitivity: A Guide to Receiver Gain Adjustment

Josh P. Peters[1*], Frank D. Sönnichsen[2], Jan-Bernd Hövener[1], Andrey N. Pravdivtsev[1*]

1. Section Biomedical Imaging, Molecular Imaging North Competence Center (MOIN CC), Department of Radiology and Neuroradiology, University Medical Center Kiel, Kiel University, Am Botanischen Garten 14, 24114, Kiel, Germany

2. Otto Diels Institute for Organic Chemistry, Kiel University, Otto Hahn Platz 4, 24118, Kiel, Germany

*Corresponding Authors: josh.peters@rad.uni-kiel.de; andrey.pravdivtsev@rad.uni-kiel.de

Contents

Signal overflow of ^{13}C-channel at the 1 T system 2

Constant signal experiment at the 9.4 T system 3

Signal and SNR sweep data at the 7 T system 4

^{1}H data at 9.4 T 7

^{2}H data at 9.4 T 9

^{13}C data at 9.4 T 11

^{15}N data at 9.4 T 13

Signal overflow of ^{13}C-channel at the 1 T system

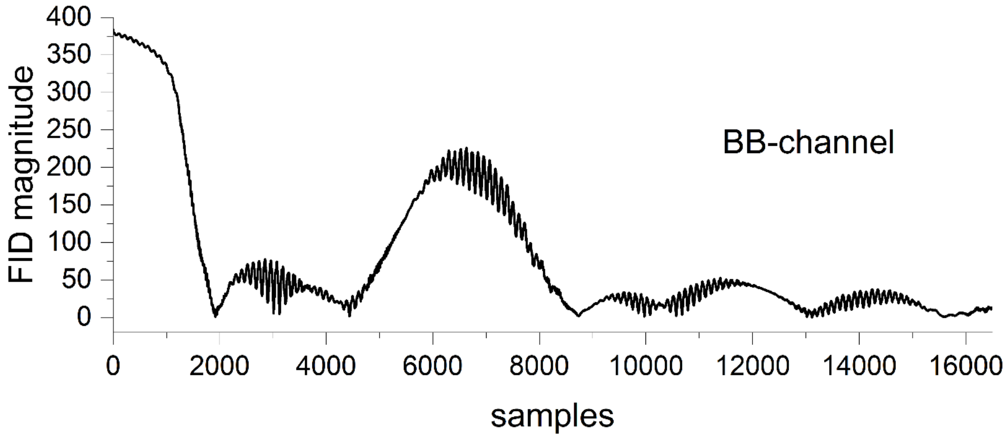

Figure S1: Magnitude ^{13}C (bottom) NMR free induction decay of hyperpolarized 1-^{13}C pyruvate (96 mM) at 1 T, where signals >≈ 380 a.u. were not adequately recorded. *Signal plotted as recorded by standard NMR spectrometer in the rotation frame of the excitation pulse; small oscillations are caused by off-resonances. The rapid decay and rising of the signal are likely caused by dynamic adjustments by the spectrometer which limits the signal to avoid overflow: This behaviour was observed in several experiments, when overflow was achieved. The signal overflow in this figure was achieved using 96 mM hyperpolarized sample with a 66° pulse and RG of 31 dB. The maximum value achieved was 384.2 a.u.*

Constant signal experiment at the 9.4 T system

Constant signal experiment as a function of RG: We did one experiment where we kept a constant $\mathrm{signal(RG)} = \mathrm{const}$. According to eq 1, this can be achieved by setting $RG \cdot \sin(\alpha)$ to a constant value. If one sets $\alpha = 90^{\circ}$ for the lowest RG of 0.25, then

$$\sin(\alpha) = \frac{0.25}{RG} \qquad \text{eq S1}$$

gives the α angle as a function of RG, providing a constant signal. Using this approach, we acquired an RG sweep with varied flip angles from 90° (RG 0.25) to 0.142° (RG 101) to keep a constant signal amplitude. The highest SNR was obtained for the combination of RG 0.25 and 90° FA and subsequently fell monotonously. This indicates that for a given signal threshold, it is best first to maximize the flip angle within the constraints of the experiment and then maximize RG as a second measure. However, maximizing the flip angle is typically not feasible, especially during a hyperpolarization experiment. In this case, the optimization procedure from the main text applies.

Note that the signal decreases from $0.85 \cdot 10^5$ a.u. to $0.60 \cdot 10^5$ a.u. between RG 0.25 to 45.2, which is consistent with the finding from the RG sweeps in **Figure 2** in the main text where the signal at lower RG values is over-proportionally high.

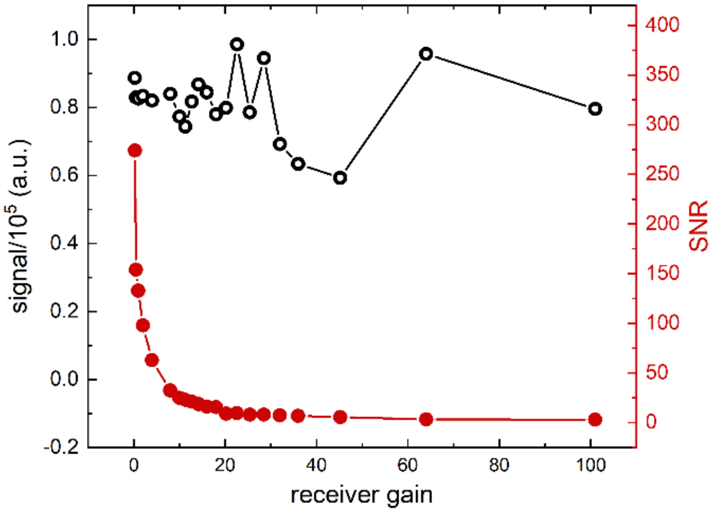

Figure S2: Constant signal experiment on the ^{15}N-sample at 9.4 T showcases the linearity of the signal and calibration of the 90° pulse. *The signal gets noisier for higher RGs, because the flipping angle gets as low as 0.142° at RG 101. It also demonstrates that for a given threshold (here signal ≈ $0.85 \cdot 10^5$ a.u.) one should maximise first the flipping angle and then RG if maximum SNR is the goal. It is apparent, similar to Figure 3-^{15}N from the main text, that the signal is higher for lower RG values if sin(α)·RG is kept constant. In theory, the signal should stay constant if the RG is linear, however here, it decreases from $0.85 \cdot 10^5$ a.u. (RG 0.25) to $0.6 \cdot 10^5$ a.u. (RG 45.2), a decrease by 30%.*

Signal and SNR sweep data at 7, 11.7, and 14.1 T

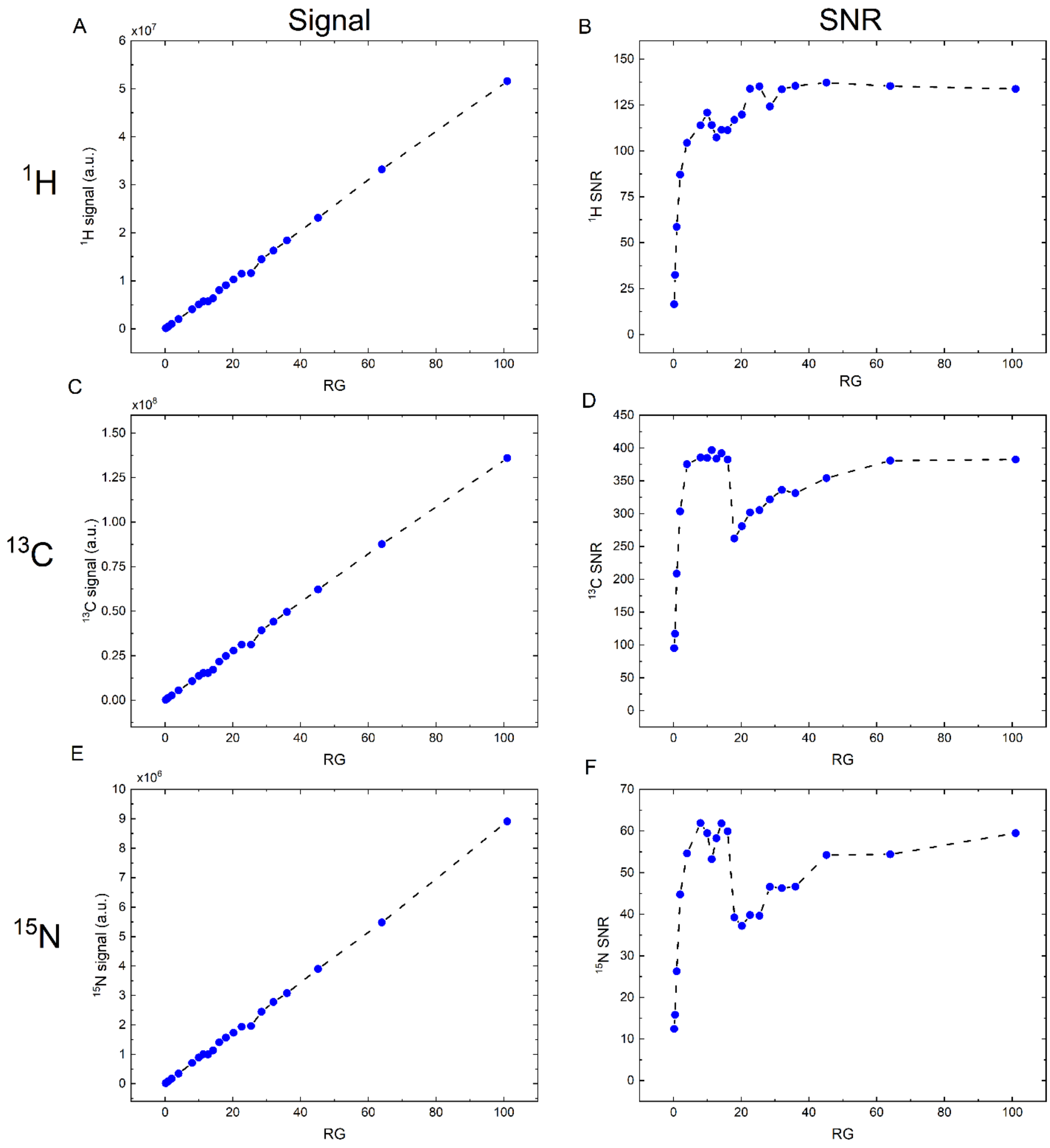

Figure S3: Signal (left column), corresponding SNR (right column), ^{1}H and ^{13}C reference samples as a function of RG at 7 T using the Avance NEO console. *The reference sample composition is given in the methods section. Note the maximum and a dip in $SNR(RG)$ after $RG = 16$ and the prolonged rate of SNR recovery for the ^{13}C sample.*

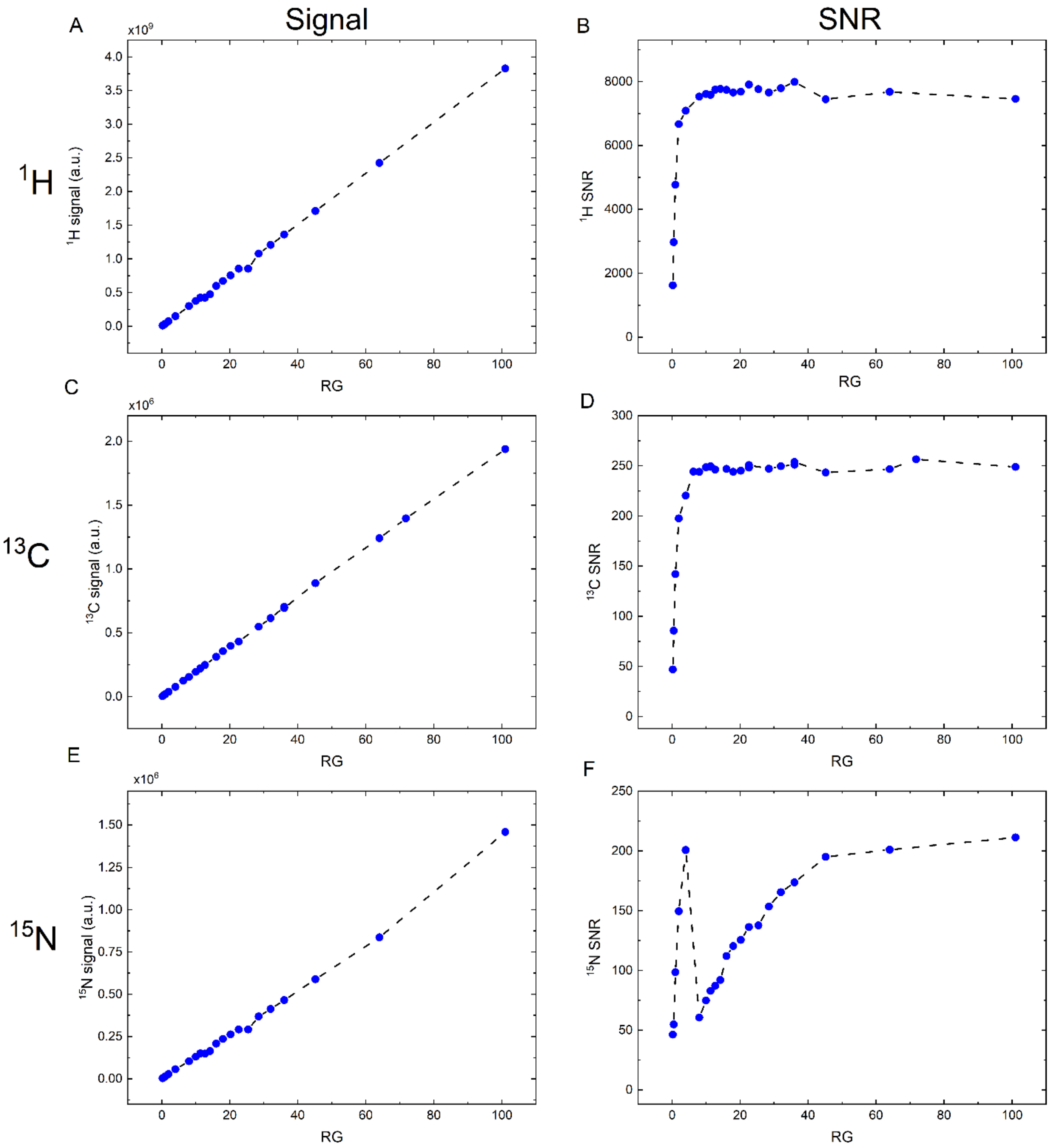

Figure S4: Signal (left column), corresponding SNR (right column), ^{1}H and ^{13}C reference samples as a function of RG at 11.7 T using the Avance NEO console. *The reference sample composition is given in the methods section. Note the maximum SNR is reached around RG 10 for both samples with a plateau thereafter.*

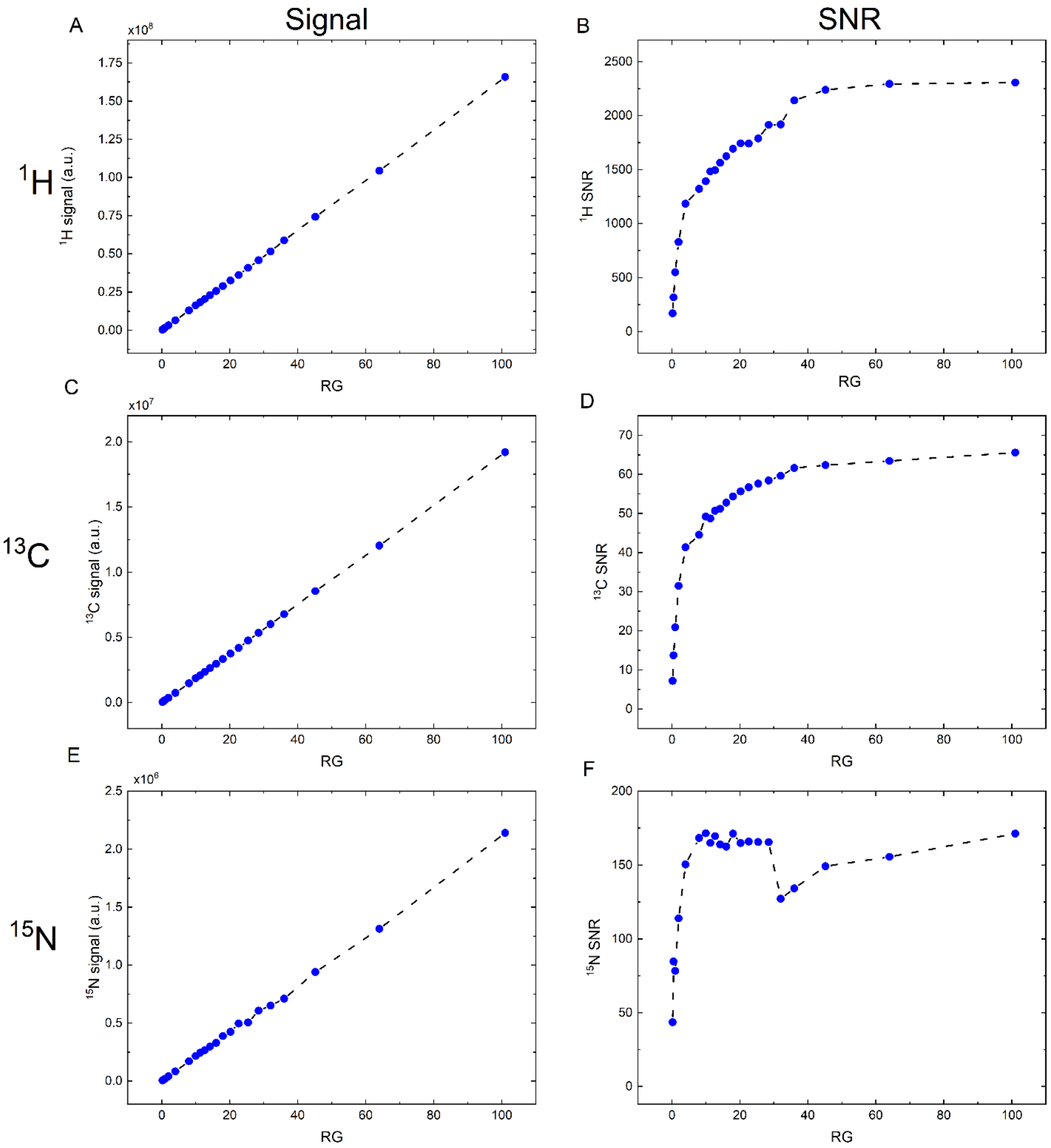

Figure S5: Signal (left), corresponding SNR (right) of ^{13}C reference sample as a function of RG at 14.1 T using a Helium cryo-probe and the Avance NEO console. *The reference sample composition is given in the methods section. Note the continuous but decelerating increase of SNR from RG 0.25 to RG 101, the maximum is reached for RG 101.*

^{1}H data at 9.4 T

Table S1: *Signal-to-noise-ratio (SNR) and maximum FID magnitude values measured with 1H sample depending on receiver gain (RG).*

RG	SNR	max FID signal (a.u.)
0.25	2.21E+03	2.13E+06
0.5	4.23E+03	4.24E+06
1	7.19E+03	8.51E+06
2	1.06E+04	1.70E+07
4	1.32E+04	3.40E+07
8	1.34E+04	6.79E+07
10	1.32E+04	8.54E+07
11.3	1.30E+04	9.48E+07
12.7	1.33E+04	1.07E+08
14.2	1.30E+04	1.19E+08
16	1.36E+04	1.34E+08
18	1.39E+04	1.49E+08
20.2	1.43E+04	1.69E+08
22.6	1.45E+04	1.89E+08
25.4	1.57E+04	2.13E+08
28.5	1.50E+04	2.38E+08
32	1.52E+04	2.68E+08
36	1.56E+04	3.00E+08
45.2	1.55E+04	3.78E+08
64	1.52E+04	5.34E+08
101	1.59E+04	8.47E+08

Data generated from analysis: /1H

++

Analysis settings:

Alpha reference: 90°

Averages reference: 8

Concentration reference: 3.456 M

Polarization reference: 0.0030981%

Concentration experiment: 111.02 M

Polarization experiment: 0.0030981%

Naximum ADC amplitude: 1.344E+09 a.u.

Extracted RG array length: 21

Extracted SNR array length: 21

Extracted signal array length: 21

Lowest RG value which yields 95% of the maximum SNR: 32

Maximum SNR in the masked plot: 459230

Corresponding RG values of max SNR: 4.89379

Corresponding flipping-angle (degrees) of max SNR: 90

Maximum product of sin(alpha) and RG with chosen concentration (111.02 M) and polarization (0.0030981%) before clipping: 4.98604

Your molar concentration in use was: 0.00343951

Maximum flip angle for RG 32 and molar concentration 0.00343951: 8.83768°

Maximum valid molar concentration for RG 32 and 90°: 0.00053728

++

^{2}H data at 9.4 T

Table S2: *Signal-to-noise-ratio (SNR) and maximum FID magnitude values measured with 2H sample depending on receiver gain (RG):*

RG	SNR	max FID signal (a.u.)
0.25	5.34E+03	2.32E+05
0.5	1.15E+04	4.62E+05
1	1.23E+04	9.22E+05
2	1.90E+04	1.84E+06
4	2.36E+04	3.68E+06
8	2.65E+04	7.31E+06
10	2.58E+04	9.23E+06
11.3	2.65E+04	1.04E+07
12.7	2.72E+04	1.16E+07
14.2	2.62E+04	1.30E+07
16	2.53E+04	1.46E+07
18	2.66E+04	1.64E+07
20.2	1.78E+04	1.85E+07
22.6	1.92E+04	2.06E+07
25.4	1.96E+04	2.32E+07
28.5	2.04E+04	2.61E+07
32	2.20E+04	2.93E+07
36	2.24E+04	3.28E+07
45.2	2.39E+04	4.14E+07
64	2.47E+04	5.85E+07
101	2.57E+04	9.25E+07

Data generated from analysis: /2H

++

Analysis settings:

Alpha reference: 90°

Averages reference: 8

Concentration reference: 2.018 M

Polarization reference: 0.000475899%

Concentration experiment: 110.42 M

Polarization experiment: 0.000475899%

Naximum ADC amplitude: 1.244E+09 a.u.

Extracted RG array length: 21

Extracted SNR array length: 21

Extracted signal array length: 21

Lowest RG value which yields 95% of the maximum SNR: 14.2

Maximum SNR in the masked plot: 1.48732E+06

Corresponding RG values of max SNR: 12.768

Corresponding flipping-angle (degrees) of max SNR: 90

Maximum product of sin(alpha) and RG with chosen concentration (110.42 M) and polarization (0.000475899%) before clipping: 24.8207

Your molar concentration in use was: 0.000525487

Maximum flip angle for RG 14.2 and molar concentration 0.000525487: 90°

Maximum valid molar concentration for RG 14.2 and 90°: 0.000919346

++

^{13}C data at 9.4 T

Table S3: *Signal-to-noise-ratio (SNR) and maximum FID magnitude values measured with ^{13}C sample depending on receiver gain (RG).*

RG	SNR	max FID signal (a.u.)
0.25	4.94E+01	2.64E+04
0.5	6.07E+01	5.14E+04
1	1.10E+02	1.03E+05
2	1.70E+02	2.05E+05
4	2.15E+02	4.00E+05
8	2.25E+02	7.91E+05
10	2.27E+02	9.76E+05
11.3	2.24E+02	1.12E+06
12.7	2.27E+02	1.25E+06
14.2	2.24E+02	1.40E+06
16	2.27E+02	1.51E+06
18	2.24E+02	1.75E+06
20.2	1.53E+02	2.03E+06
22.6	1.66E+02	2.28E+06
25.4	1.79E+02	2.50E+06
28.5	1.83E+02	2.93E+06
32	1.89E+02	3.22E+06
36	1.97E+02	3.56E+06
45.2	2.06E+02	4.41E+06
64	2.13E+02	6.42E+06
101	2.21E+02	9.83E+06

Data generated from analysis: /13C

++

Analysis settings:

Alpha reference: 90°

Averages reference: 2

Concentration reference: 1.308 M

Polarization reference: 0.00077917%

Concentration experiment: 0.09 M

Polarization experiment: 35%

Naximum ADC amplitude: 1.244E+09 a.u.

Extracted RG array length: 21

Extracted SNR array length: 21

Extracted signal array length: 21

Lowest RG value which yields 95% of the maximum SNR: 8

Maximum SNR in the masked plot: 60998.8

Corresponding RG values of max SNR: 9.40909

Corresponding flipping-angle (degrees) of max SNR: 5

Maximum product of sin(alpha) and RG with chosen concentration (0.09 M) and polarization (35%) before clipping: 4.1353

Your molar concentration in use was: 0.0315

Maximum flip angle for RG 8 and molar concentration 0.0315: 30.5513°

Maximum valid molar concentration for RG 8 and 90°: 0.182103

++

^{15}N data at 9.4 T

Table S4: *Signal-to-noise-ratio (SNR) and maximum FID magnitude values measured with ^{15}N sample depending on receiver gain (RG).*

RG	SNR	max FID signal (a.u.)
0.25	4.52E+01	4.03E+03
0.5	5.73E+01	7.29E+03
1	9.91E+01	1.42E+04
2	1.60E+02	2.69E+04
4	1.91E+02	5.32E+04
8	2.00E+02	1.02E+05
10	2.03E+02	1.31E+05
11.3	2.08E+02	1.48E+05
12.7	2.05E+02	1.65E+05
14.2	2.08E+02	1.79E+05
16	2.05E+02	2.05E+05
18	2.03E+02	2.23E+05
20.2	1.39E+02	2.63E+05
22.6	1.48E+02	2.92E+05
25.4	1.54E+02	3.35E+05
28.5	1.62E+02	3.64E+05
32	1.72E+02	4.08E+05
36	1.76E+02	4.75E+05
45.2	1.89E+02	5.71E+05
64	1.90E+02	8.32E+05
101	2.06E+02	1.26E+06

Data generated from analysis: /15N

++

Analysis settings:

Alpha reference: 90°

Averages reference: 16

Concentration reference: 2.059 M

Polarization reference: 0.00031414%

Concentration experiment: 0.04 M

Polarization experiment: 15%

Naximum ADC amplitude: 1.244E+09 a.u.

Extracted RG array length: 21

Extracted SNR array length: 21

Extracted signal array length: 21

Lowest RG value which yields 95% of the maximum SNR: 8

Maximum SNR in the masked plot: 33507.7

Corresponding RG values of max SNR: 14.1814

Corresponding flipping-angle (degrees) of max SNR: 10

Maximum product of sin(alpha) and RG with chosen concentration (0.04 M) and polarization (15%) before clipping: 107.151

Your molar concentration in use was: 0.006

Maximum flip angle for RG 8 and molar concentration 0.006: 90°

Maximum valid molar concentration for RG 8 and 90°: 0.452103

++
